# Atypical Spontaneous Cecal Perforation During Redo Kidney Transplant Post Heart‐Kidney Transplantation

**DOI:** 10.1155/crit/4653334

**Published:** 2026-10-25

**Authors:** Valeria Ripa, Stuart R. Geffner, Matthew Hanlon, Matthew G. Rosenzweig, Harry Sun

**Affiliations:** ^1^ Renal and Pancreas Transplant Division Cooperman Barnabas Medical Center Livingston New Jersey USA

## Abstract

Colonic perforation postrenal transplantation is rare (0.5%–2.3% incidence) and often associated with sigmoid diverticulitis, with reported mortality rates of 25%–50%. Right‐sided spontaneous colonic perforation is particularly uncommon. We describe a 44‐year‐old male with a history of combined heart–kidney transplantation who developed rapid‐onset cecal perforation following repeat deceased donor kidney transplantation. Despite early return of bowel function, an aggressive bowel regimen, and minimal opioid exposure, he developed acute abdominal distention, nausea, and obstipation on postoperative Day 5. Although he remained afebrile without leukocytosis or peritoneal signs, imaging demonstrated marked colonic dilatation and pneumoperitoneum, prompting emergent surgical exploration. Isolated cecal perforation was identified and successfully managed with right hemicolectomy, primary anastomosis, and diverting ileostomy. The patient recovered with preserved allograft function. This case underscores the potential for atypical and insidious gastrointestinal complications in patients with prolonged and intensified immunosuppression and highlights the importance of early imaging and prompt surgical intervention. Heightened clinical suspicion is essential as retransplantation and multiorgan transplant populations continue to expand.

## 1. Introduction

Advances in solid organ transplantation and surgical techniques over the past two decades have significantly improved graft survival and reduced patient mortality [1]. Concurrently, increasing numbers of combined solid organ transplant recipients and patients undergoing second or third kidney transplantation have created a growing population with prolonged immunosuppression exposure and complex perioperative risk profiles [2, 3].

Colonic perforation after renal transplantation remains a rare but devastating complication, associated with high morbidity and mortality [4]. Most reported colonic complications in this population stem from diverticular disease, predominantly affecting the left colon, likely due to the higher prevalence of diverticulosis and chronic constipation in patients on chronic dialysis [5–7]. Nonobstructive colonic dilatation, also known as Ogilvie′s syndrome (acute colonic pseudo‐obstruction), has been reported only rarely following renal transplantation, despite its association with various medical and surgical conditions [8, 9].

This case report describes an unusual presentation of rapid‐onset nonobstructive colonic dilatation leading to spontaneous cecal perforation in a patient with a prior combined heart‐kidney transplant who underwent a second kidney transplant. No mechanical obstruction, diverticulitis, or infectious colitis was identified. Notably, the event occurred despite return of bowel function, an aggressive bowel regimen, and minimal opioid exposure.

## 2. Case Report

A 44‐year‐old male with end‐stage renal disease (ESRD) and a complex cardiac and transplant history presented for deceased donor kidney transplantation in October 2025 at Cooperman Barnabas Medical Center. His medical and surgical history included morbid obesity with prior laparoscopic adjustable gastric banding, insulin‐dependent Type 2 diabetes mellitus, hyperlipidemia, obstructive sleep apnea, seizure disorder, and nonischemic cardiomyopathy diagnosed at age 18.

His cardiomyopathy progressed over time, requiring implantable cardioverter‐defibrillator placement at age 27, with device replacement 3 years later due to malfunction. In his mid‐30s, he developed advanced heart failure refractory to medical therapy and failed home milrinone infusion. At age 36, he underwent implantation of a HeartMate II left ventricular assist device as bridge‐to‐transplant and was subsequently listed for heart transplantation as status 1B.

Chronic kidney disease progressed to ESRD attributed to cardiorenal syndrome in the setting of chronic hemodynamic instability and right ventricular dysfunction. Hemodialysis was initiated.

At age 38, he underwent orthotopic heart transplantation with a simultaneous kidney transplant from a hepatitis C–positive donor. The postoperative course was notable for a temporary requirement for enteral feeding support. The renal allograft subsequently demonstrated primary nonfunction, whereas cardiac allograft function remained well preserved. Kidney biopsy showed extensive ischemic injury with scarring and necrosis. He received direct‐acting antiviral therapy for donor‐derived hepatitis C and remained hepatitis C RNA negative. He was relisted for kidney transplantation in his early 40s. The patient remained on single‐agent immunosuppression with tacrolimus. As part of his evaluation for repeat kidney transplantation, colonoscopy performed approximately 2 months before transplantation was normal. A chronological summary of the patient′s clinical and transplant course is provided in Table 1.

**Table 1:** Timeline of the patient′s clinical and transplant course.

Date	Age (years)	Clinical event
1999	18	Diagnosed with nonischemic cardiomyopathy.
2006	20	Laparoscopic adjustable gastric band placement.
2008	27	Implantable cardioverter‐defibrillator placed.
2011	30	Implantable cardioverter‐defibrillator replaced because of device malfunction.
2017	36	Progression to advanced heart failure refractory to medical therapy; HeartMate II left ventricular assist device implanted as a bridge to transplantation.
10/2019	38	Chronic kidney disease progressed to ESRD secondary to cardiorenal syndrome; hemodialysis initiated.
10/2019	38	Simultaneous orthotopic heart and kidney transplantation. Cardiac allograft function remained stable without rejection; renal allograft developed primary nonfunction, with continued dialysis dependence.
9/20/2022	40	Relisted for kidney transplantation.
10/23/2025	44	Repeat deceased donor kidney transplantation.
10/24/2025 (POD 1)	44	Delayed graft function. Urine output 100 mL/day; serum creatinine 7.2 mg/dL; BUN 28.6 mg/dL; WBC 9.1 × 10^9^/L.
10/25/2025 (POD 2)	44	Passage of flatus. Urine output 313 mL/day; serum creatinine 8.6 mg/dL; BUN 46.4 mg/dL; WBC 6.7 × 10^9^/L.
10/26/2025 (POD 3)	44	Continued passage of flatus with improving urine output to 900 mL/day; serum creatinine 8.5 mg/dL; BUN 55.4 mg/dL; WBC 4.5 × 10^9^/L.
10/27/2025 (POD 4)	44	First bowel movement. Urine output 950 mL/day; serum creatinine 7.3 mg/dL; BUN 63.3 mg/dL; WBC 4.5 × 10^9^/L.
10/28/2025 (POD 5)	44	Acute abdominal distention, nausea, and obstipation. Imaging demonstrated marked colonic dilatation and pneumoperitoneum. Emergent exploratory laparotomy revealed focal cecal perforation with fecal peritonitis. The patient underwent right hemicolectomy with primary anastomosis and diverting ileostomy; the gastric band removal. Urine output 1020 mL/day; serum creatinine 5.4 mg/dL; BUN 63.9 mg/dL; WBC 7.1 × 10^9^/L.
10/29/2025 (POD 6)	44	Hemodynamically stable following laparotomy. Urine output 1350 mL/day; serum creatinine 4.2 mg/dL; BUN 61.8 mg/dL; WBC 11.0 × 10^9^/L.
10/30/2025 (POD 7)	44	Return of ostomy function; diet advanced. Urine output 1380 mL/day; serum creatinine 3.6 mg/dL; BUN 60.7 mg/dL; WBC 9.3 × 10^9^/L.
10/31/2025 (POD 8)	44	Tolerating diet with continued clinical recovery. Urine output 1380 mL/day; serum creatinine 3.1 mg/dL; BUN 58.1 mg/dL; WBC 9.6 × 10^9^/L.
11/06/2025 (POD 14)	44	Discharged home. Urine output 1770 mL/day; serum creatinine 2.1 mg/dL; BUN 39.2 mg/dL; WBC 11.6 × 10^9^/L.
1/21/2026 (3‐month follow‐up)	44	Clinically recovered and doing well, with stable kidney allograft function; serum creatinine 1.6 mg/dL.
3/12/2026	45	Elective ileostomy reversal; kidney allograft function remained stable, with serum creatinine 1.3 mg/dL.
4/23/2026 (6‐month follow‐up)	45	Clinically well following ileostomy reversal, with stable kidney allograft function; serum creatinine 1.4 mg/dL.
8/20/2026 (last follow‐up)	45	Clinically doing well, serum creatinine 1.60 mg/dL.

Abbreviations: BUN, blood urea nitrogen; ESRD, end‐stage renal disease; POD, postoperative day; WBC, white blood cell count.

At age 44, he underwent repeat deceased donor kidney transplantation with a negative virtual crossmatch. The donor was a 47‐year‐old female with donation after circulatory death due to cerebrovascular accident. Kidney donor profile index was 41%. Procurement biopsy demonstrated minimal chronic changes (2% glomerulosclerosis). The kidney allograft was obtained through the legally regulated United States organ donation system, coordinated by Gift of Life Donor Program (Philadelphia, Pennsylvania). Donation authorization and organ recovery arrangements were managed by the organ procurement organization. The authors′ transplant team did not participate in organ recovery.

Induction immunosuppression consisted of thymoglobulin (total dose 6 mg/kg) and intravenous methylprednisolone 500 mg, followed by maintenance immunosuppression with mycophenolate mofetil, a prednisone taper, and tacrolimus with a target trough level of 8–10 ng/mL. The kidney, with single artery and vein, was implanted in the left retroperitoneum and anastomosed to the external iliac vessels in standard fashion without intraoperative complication.

The postoperative course was notable for delayed graft function with steadily improving urine output, increasing from minimal volumes on postoperative Day 1 to over 1 L/day by postoperative Day 5, and gradual improvement in serum creatinine. Pain was managed with a multimodal regimen including a transversus abdominis plane block, scheduled acetaminophen, gabapentin, and methocarbamol, with minimal opioid use.

Bowel function began to return on postoperative Day 2, when the patient started to pass flatus consistently. He remained on a three‐agent bowel regimen (docusate, senna, and polyethylene glycol) and had a bowel movement on postoperative Day 4. Throughout this period, vital signs remained stable, with no fever or chills. Abdominal examination was benign, without tenderness or peritoneal signs.

On postoperative Day 5, the patient developed acute abdominal distention, nausea, and obstipation. There was no leukocytosis at the time of clinical deterioration; the WBC count was 7.1 × 10^9^/L. Abdominal radiography demonstrated multiple dilated loops of small bowel with marked gaseous distention of the ascending colon to 14 cm (Figure 1). Noncontrast computed tomography revealed a large volume of free intraperitoneal air, fecal stasis within the right colon, and mild pericolic inflammatory changes, without an intra‐abdominal fluid collection (Figure 2).

**Figure 1:**
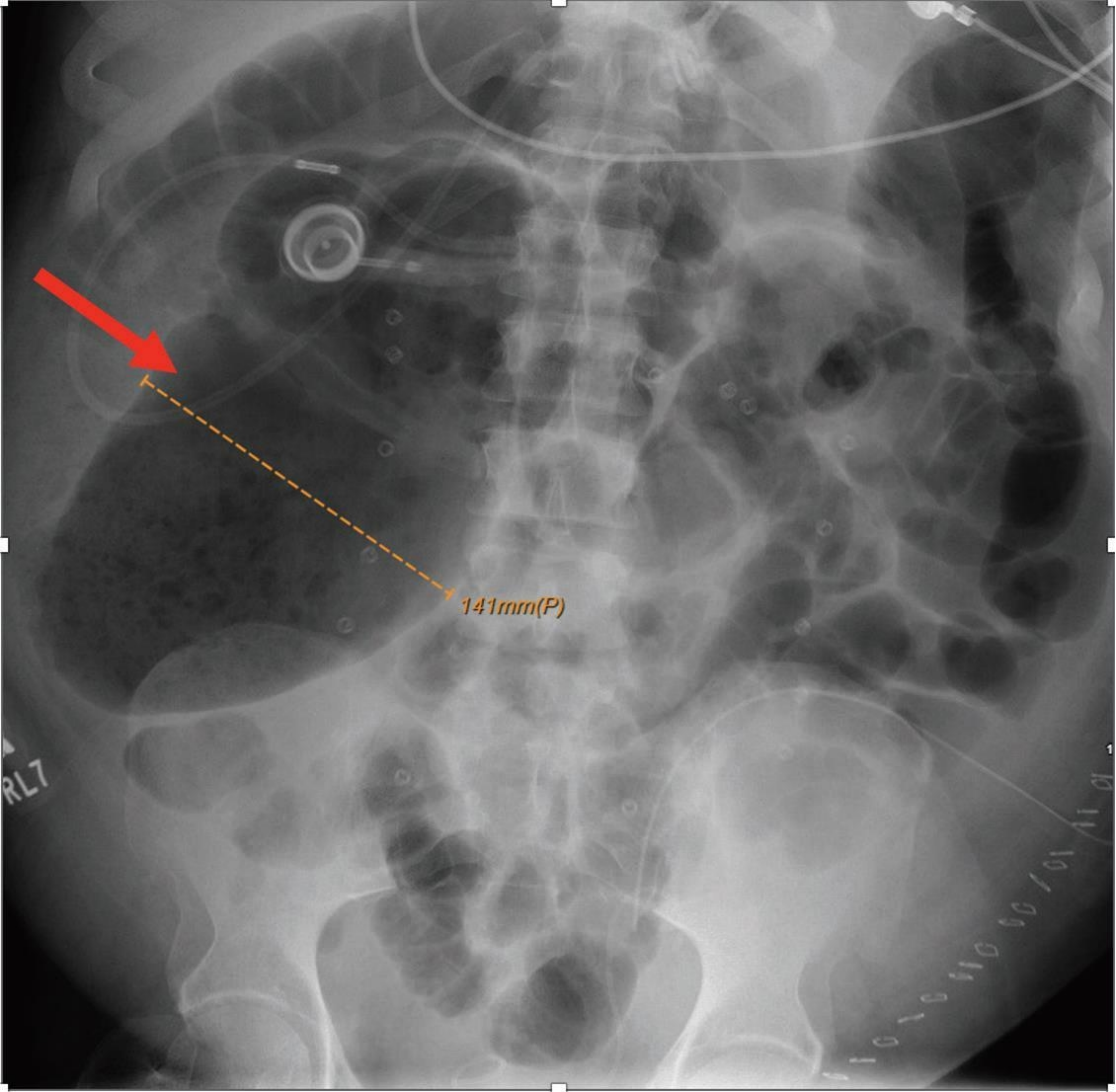
Abdominal radiograph obtained on postoperative Day 5 following repeat kidney transplantation. Marked gaseous dilatation of the right colon is present, with the ascending colon/cecum measuring approximately 14 cm in maximal diameter. The finding developed in the setting of acute abdominal distention, nausea, and obstipation.

**Figure 2:**
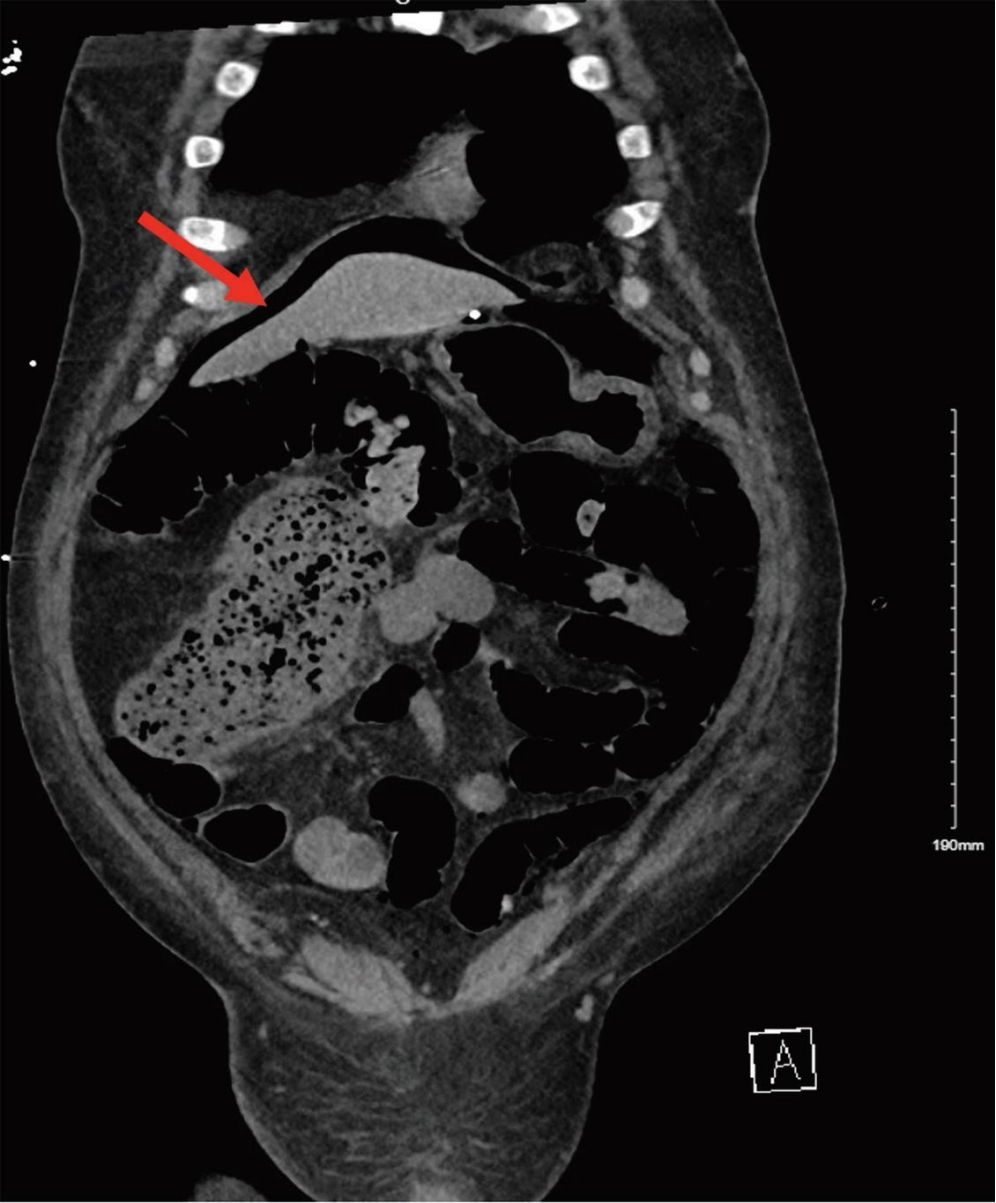
Coronal CT of the abdomen and pelvis obtained on postoperative Day 5. Marked right‐colonic distention with fecal loading is accompanied by extensive free intraperitoneal air (red arrow), consistent with hollow‐viscus perforation.

He was taken emergently for exploratory laparotomy. Intraoperative findings revealed a focal perforation of the cecum with fecal peritonitis, without evidence of diverticulitis, mechanical obstruction, or infectious colitis or additional bowel injury. A right hemicolectomy with side‐to‐side functional anastomosis was performed, and an ileostomy was created. The gastric band was removed due to gross contamination and the patient′s immunosuppressed state.

The postoperative course following laparotomy was uncomplicated. The patient remained hemodynamically stable, with continued improvement in kidney allograft function. Maintenance immunosuppression was continued without interruption. Although the patient remained nil per os, mycophenolate mofetil and corticosteroids were administered intravenously, and tacrolimus was continued via nasogastric tube. Ostomy function returned appropriately, diet was advanced, and serum creatinine progressively declined. As oral intake resumed, maintenance immunosuppression was transitioned back to oral administration. He was discharged home on postoperative Day 14 with a functioning ileostomy and a serum creatinine of 2.1 mg/dL.

Pathologic examination of the resected colon demonstrated perforation with necrosis, ulceration, acute inflammation, edema, and hemorrhage, with two benign lymph nodes identified. At 3‐month follow‐up, the patient had clinically recovered and kidney allograft function remained stable, with a serum creatinine of 1.6 mg/dL. He subsequently underwent elective ileostomy reversal 5 months after transplantation, with an uncomplicated postoperative recovery and preserved allograft function. At the most recent follow‐up, approximately 10 months after transplantation, he remained clinically well with stable kidney allograft function.

## 3. Discussion

In the United States, ESRD affects over 808,000 individuals, approximately 68% of whom are on dialysis and 32% have a functioning kidney transplant; in 2023, a record 28,142 kidney transplants were performed [10]. Cumulative transplant volumes continue to grow, with advancements in immunosuppression and surgical techniques enabling longer patient survival and increasing numbers of individuals requiring second or third kidney transplants [1, 3, 10].

Combined heart‐kidney transplants have risen substantially as well (e.g., from 76 in 2012 to 421 in 2023), and retransplantation is increasingly common in sensitized patients or those with failed prior grafts [11]. These patients often face prolonged immunosuppression exposure, high sensitization rates, and the need for intensified induction and maintenance regimens [12]. These factors increase perioperative risk and may lead to atypical or masked presentations of postoperative complications [13]. This is especially relevant in patients who return to dialysis while remaining immunosuppressed after graft failure, where chronic steroid exposure and blunted inflammatory responses can delay recognition [13].

Gastrointestinal complications, including colonic perforation, are uncommon but potentially life‐threatening after kidney transplantation, with reported incidences ranging from 0.5% to 2.3% [14–17]. The majority of reported cases involve the sigmoid colon and are most frequently associated with diverticular disease, which represents the leading etiology and has been reported in up to 75% of cases in some series [15]. Other described causes include colonic ischemia, infectious colitis (e.g., cytomegalovirus, tuberculosis, or histoplasmosis), medication‐related injury (such as sodium polystyrene sulfonate), fecal impaction, and colonic ulceration (Figure 3) [4, 15, 17, 18]. A substantial proportion of cases are reported without an identifiable underlying cause [18]. Identified risk factors include deceased donor transplantation, higher body mass index, prior diverticular disease, and intensive early postoperative immunosuppression [19, 20]. However, contemporary data examining the relationship between specific modern immunosuppressive regimens and colonic perforation remain limited [21].

**Figure 3:**
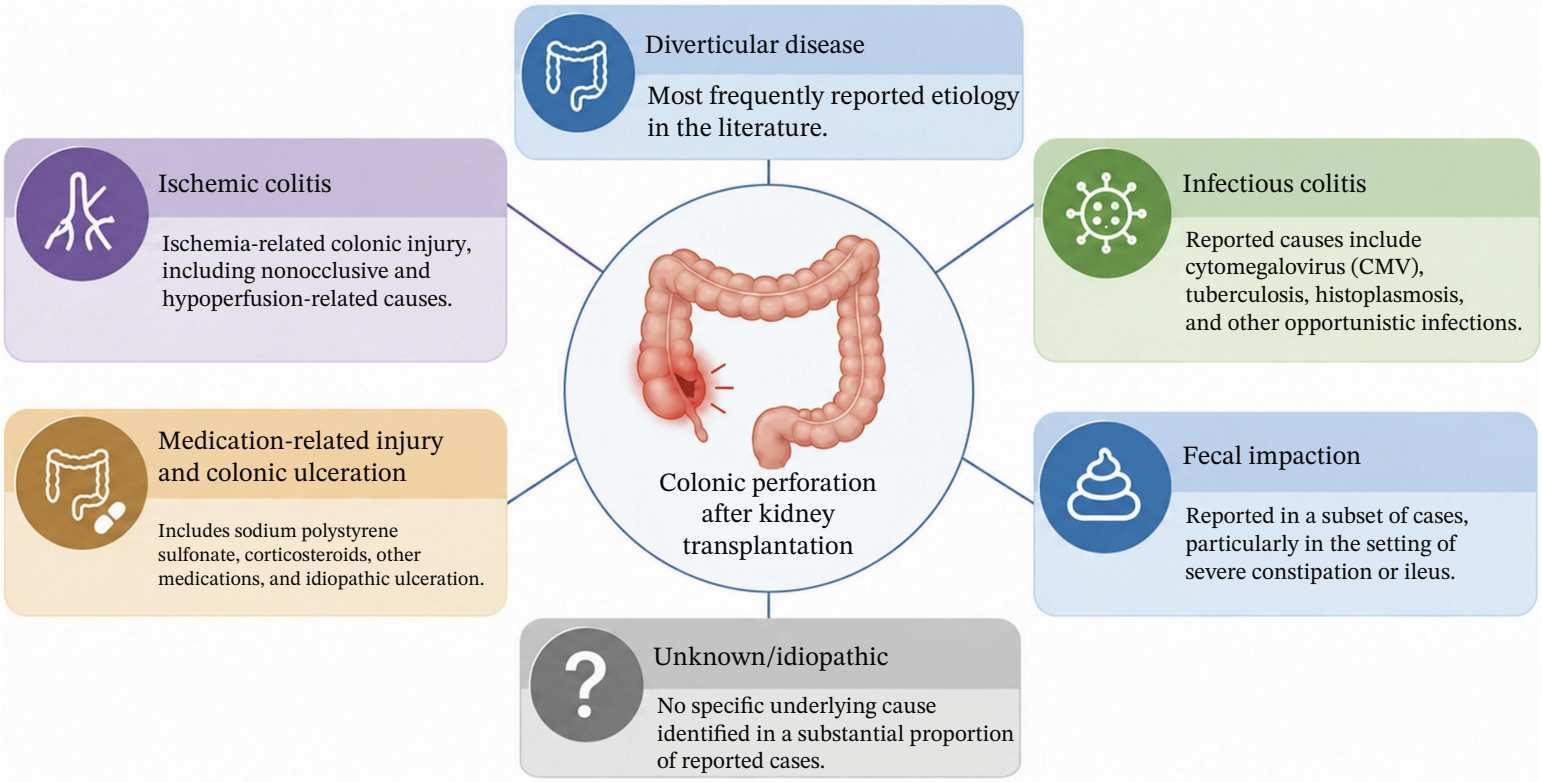
Reported etiologies of colonic perforation after kidney transplantation.

Clinical presentation is frequently subtle, with mild abdominal discomfort without overt peritonitis, whereas laboratory findings may be nonspecific [13, 21]. Prompt diagnosis and aggressive surgical intervention—typically bowel resection with diversion—are critical, with mortality historically ranging from 25% to over 50% in older reports, though lower with early recognition [14, 17].

Our patient underwent repeat kidney transplantation after a prior heart–kidney transplant; the cardiac allograft remained intact, whereas the initial kidney graft developed primary nonfunction. He had prolonged exposure to tacrolimus‐based immunosuppression with steroid avoidance. Bowel function had returned postoperatively with strict adherence to an aggressive bowel regimen routinely employed at our center. This regimen included stool softeners and osmotic laxatives, with escalation to suppositories or enemas if needed. To minimize constipation and fecal stasis, a multimodal pain management protocol was implemented with minimal opioid use reserved for breakthrough pain. Despite these measures, the patient developed abrupt nausea, abdominal distention, cessation of flatus, and rapid progression to marked cecal dilation and perforation within 24 h. His abdominal examination remained largely benign, and he never developed fever, chills, or leukocytosis at the time of clinical deterioration.

The most likely precipitating etiology was nonobstructive colonic dilation (Ogilvie syndrome), as suggested by radiographic findings. The pathogenesis of acute colonic pseudo‐obstruction remains incompletely understood and is thought to involve dysregulation of autonomic control of colonic motility [22]. Similar cases have been reported in renal transplant recipients, frequently in association with poor allograft function [4, 23]. In our patient, however, allograft function was progressively improving, with no evidence of rejection or major electrolyte disturbance at the time of perforation. Despite the absence of these previously described associations, rapid colonic dilation provided a plausible pathway to cecal ischemia and subsequent perforation.

Progressive colonic distension can result in cecal rupture, as explained by Laplace′s law, which relates wall tension to the radius of a hollow viscus [24]. Increasing intraluminal pressure may compromise bowel wall perfusion, ultimately resulting in ischemia and necrosis [25]. Pathologic examination in our patient revealed cecal perforation with necrosis, ulceration, acute inflammation, edema, and hemorrhage, supporting a mechanism of ischemia secondary to colonic distention.

Early surgical intervention is critical when progressive colonic distention is associated with ischemia or impending rupture [24]. Although successful colonoscopic decompression has been reported in uncomplicated acute colonic pseudo‐obstruction, the presence of pneumoperitoneum and fecal peritonitis in our patient required emergent operative management [24].

Because the precipitating mechanism of acute colonic dilation remains uncertain, specific preventive strategies cannot be established from this case. Standard measures, including a standing bowel regimen, early ambulation, and opioid‐minimizing multimodal analgesia, were already employed. The principal opportunity for risk mitigation may therefore lie in early recognition of new abdominal distention or cessation of bowel function and a low threshold for abdominal imaging before progression to ischemia and necrosis.

In summary, as retransplantation increases in an aging, complex population, clinicians must remain alert for rare and atypical surgical complications. This case demonstrates an unusual presentation of acute cecal perforation following kidney retransplantation, with rapid clinical progression despite an initially benign clinical presentation. A high index of suspicion when new abdominal symptoms develop may facilitate earlier recognition and intervention. Further studies evaluating preventive strategies in multiorgan retransplant recipients may help reduce the occurrence of such events.

## Funding

No funding was received for this manuscript.

## Ethics Statement

This study was conducted in accordance with institutional ethical standards and the Declaration of Helsinki.

## Consent

Written informed consent was obtained from the patient for publication of this case report and any accompanying images.

## Conflicts of Interest

The authors declare no conflicts of interest.

## Data Availability

The data that support the findings of this study are available on request from the corresponding author. The data are not publicly available due to privacy or ethical restrictions.
